# A Behavior-Based Quality Improvement Initiative for Intensive Care Unit Calmness: A Single-Center Before-After Evaluation

**DOI:** 10.7759/cureus.116331

**Published:** 2026-09-15

**Authors:** Dhivahar Ganesan, Raghunath Doraiswamy, Athira Rajkumar

**Affiliations:** 1 Critical Care Medicine, Gastroenterology Medical Center and Hospital (GEM Hospital), Coimbatore, IND

**Keywords:** human factors, intensive care unit, noise, nursing, quality improvement, squire 2.0

## Abstract

Introduction

Intensive care units (ICUs) are acoustically and cognitively demanding environments in which modifiable behavioral factors, unnecessary verbal communication, non-actionable alarms, disorganized workflow, and unstructured handovers contribute substantially to noise burden and cognitive overload for nursing staff. Published Indian ICU literature on this problem remains predominantly measurement-focused. We developed the CALMER bundle, a six-component, no-capital-expenditure behavioral intervention (communication discipline, alarm discipline, leadership modeling, mindful workflow and equipment handling, emotional reset, and reinforcement). We evaluated its feasibility and perceived impact in an Indian tertiary ICU.

Materials and methods

This was a single-center, uncontrolled, before-after quality improvement study conducted in a 20-bed mixed medical-surgical ICU at GEM Hospital, Coimbatore, India, reported per the Standards for Quality Improvement Reporting Excellence (SQUIRE) 2.0 guidelines. The primary outcome, composite perceived ICU calmness, was assessed using a retrospective paired then-now 11-item questionnaire (five-point Likert scale; Cronbach's alpha 0.831), administered to all 40 nursing staff with complete-case analysis in 28 nurses (28/40, 70%). Secondary outcomes included a six-item work environment score (same 28 nurses) and a post-intervention adapted NASA Task Load Index (NASA-TLX) workload assessment (n = 40). We assessed patient/surrogate perception cross-sectionally post-intervention (n = 100). We analyzed paired ordinal data using the Wilcoxon signed-rank test, with effect size r and 95% confidence intervals (Fisher's z-transformation).

Results

Composite ICU calmness improved from a pre-intervention median of 2.0 (IQR: 1.75-2.0) to 4.0 (IQR: 4.0-4.0) (p < 0.001; r = 0.910; 95% CI: 0.813-0.958). All four calmness domains improved significantly (r = 0.842-0.874). Twenty-six of 28 nurses (92.9%) met a prespecified ≥2-point responder threshold; none declined. The work environment score improved from 2.25 to 4.0 (p < 0.001; r = 0.874), with all six secondary items improving significantly (r = 0.751-0.872). Post-intervention workload was at the scale midpoint (NASA-TLX median: 3.0/5). Patient/surrogate perceptions were favorable across all five domains (median: 5.0/5); as a post-intervention-only assessment, these are reported descriptively without a baseline comparator. A later-phase implementation-fidelity audit (141 shift-level audits, May 7 to June 30, 2026) showed 82.9% overall compliance (highest: leadership modeling 91.8%; lowest: emotional reset 76.2%). No alarm-related sentinel events occurred.

Conclusions

A zero-capital-expenditure, behavior-based bundle was associated with large, statistically significant improvements in nurse-perceived ICU calmness and work environment in an Indian tertiary ICU, alongside a post-intervention workload rating at the scale midpoint. Unblinded, investigator-administered assessment and the absence of objective acoustic data are important limitations. Multicenter validation with objective noise measurement and independent data collection is needed before generalizing these findings.

## Introduction

Nature and significance of the problem

Few environments in modern healthcare are as acoustically and cognitively demanding as the intensive care unit (ICU). Noise levels here routinely exceed World Health Organization recommended limits, originally specified for general hospital patient-room environments rather than ICU settings specifically, of 35 dB(A) by day and 30 dB(A) at night.

This excessive noise is not purely an architectural problem. A substantial body of evidence points instead to modifiable behavioral factors, unnecessary verbal communication, non-actionable alarm burden, disorganized workflow clustering, and unstructured shift handovers as primary contributors to acoustic overload and cognitive disruption [1-5].

For ICU nurses, the situation takes a compounding toll. Frequent interruptions during medication administration, alarm desensitization, and sustained emotional demands together are associated with cognitive overload that may impair clinical focus, increase error risk, and contribute to professional burnout [4,5]. From the patient perspective, ICU noise and sensory disruption are among the most consistently reported sources of distress and have been associated with sleep deprivation, delirium, heightened anxiety, and impaired recovery [3].

Beyond acoustic burden, ICU calmness may represent a broader human-factor construct reflecting the interaction of environmental, behavioral, cognitive, and organizational influences, one that is not fully captured by decibel measurement alone. In this study, perceived ICU calmness is operationally defined as nursing staff's subjective rating of the ICU environment across four domains: environmental calmness, cognitive focus, communication quality, and workflow coordination, captured by the 11-item study instrument (Appendices); it is treated as the primary outcome construct, distinct from noise burden and cognitive overload, which are conceptualized as modifiable mechanisms hypothesized to drive poor calmness rather than as synonymous outcomes [6]. This noise-to-cognitive-overload-to-calmness pathway is a hypothesized conceptual framework informing the study's design and interpretation, not demonstrated causality; validity evidence for the calmness instrument is limited to internal consistency (Cronbach's alpha), as noted in the limitations.

To our knowledge, no prior Indian adult ICU study in the indexed literature has evaluated a structured multicomponent behavior bundle with perceived calmness as the primary outcome. This study extends existing Indian ICU noise research, which has focused predominantly on acoustic measurement, by positioning calmness culture as both the intervention target and the measured endpoint. The novelty of CALMER lies not in using a bundle per se, but in selecting behavioral calmness as the primary evaluative endpoint, measured through a nurse-reported instrument, in an Indian adult ICU setting. Low-resource, behavior-focused, scalable interventions that can be implemented without additional capital expenditure are clearly needed in resource-constrained settings [1,2].

Available knowledge

Existing ICU noise reduction strategies fall into four broad categories. Environmental and technological interventions, including acoustic panels, single-occupancy rooms, and architectural redesign, have demonstrated measurable reductions in ambient noise levels but require significant institutional investment [1,2]. Behavioral interventions have emerged as particularly promising given their low cost and transferability: Konkani et al. demonstrated that a behavior-focused noise reduction program targeting staff communication and alarm management significantly reduced perceived noise burden and improved staff satisfaction [1], and Tronstad et al. reported measurable improvements in sound levels and patient comfort following a structured sound reduction bundle in an Australian ICU [2]. Multicomponent interventions in Indian critical care remain limited to a neonatal multimodal noise-reduction bundle [7]; to our knowledge, no prior Indian adult ICU study has evaluated a broader, staff-perception-based behavioral calmness bundle.

Effects on Staff

The interruption science literature supports the mechanistic plausibility of behavior-based calmness interventions, linking interruptions and alarm burden to cognitive overload among nursing staff [4,5].

Effects on Patients

Darbyshire and Young confirmed that ICU sound levels substantially and consistently exceed WHO-recommended thresholds [3], and an Indian adult ICU noise assessment and source-mapping study has similarly documented elevated noise exposure [8], though without evaluating a corresponding intervention.

Evidence Gap and Rationale for CALMER

The Systems Engineering Initiative for Patient Safety (SEIPS) model underscores that ICU work system design must be addressed holistically to achieve sustained behavioral change [6], and Pronovost's translating evidence into practice framework supports bundled interventions, with leadership engagement as an essential implementation driver [9]. To our knowledge, CALMER is the first structured, behavior-based ICU calmness bundle reported in an Indian adult ICU, extending existing Indian ICU noise research, which has focused predominantly on acoustic measurement [8], by positioning calmness culture as both the intervention target and the measured endpoint.

Rationale for the CALMER bundle

The CALMER bundle was developed in response to observed behavioral and cultural drivers of ICU noise and cognitive disruption at a 20-bed tertiary mixed medical-surgical ICU. Informal pre-implementation staff feedback and bedside observations identified low perceived calmness, high work strain, alarm fatigue, and unstructured communication patterns as the dominant concerns. These findings informed a theory-driven, multicomponent behavioral bundle designed to target identified drivers using low-resource, implementable strategies consistent with Indian ICU operational realities [6,10,11].

The CALMER acronym encapsulates six interdependent behavioral domains: communication discipline (C), alarm discipline (A), leadership modeling (L), mindful workflow and equipment handling (M), emotional reset (E), and reinforcement (R). This framework is grounded in human factors theory, teamwork science, and the principles of sustainable quality improvement [6,9,12,13]. CALMER is a novel bundle developed de novo for this study; no validated behavioral bundle targeting ICU noise and calmness exists in the published Indian adult ICU literature. This study is the first formal evaluation, and multicenter validation is a necessary next step.

Retrospectively, the six CALMER domains map onto the SEIPS work-system components, offering an interpretive rather than a prospectively applied theoretical lens [6]. Emotional reset corresponds to the person component, addressing the individual nurse's psychological capability and cognitive endurance. Mindful workflow and equipment handling correspond to tasks, changing how nurses carry out bedside care activities. Alarm discipline corresponds to the environment component, modifying the acoustic and technological interface. Leadership modeling and reinforcement correspond to the organization component, shaping culture and management structure. Communication discipline spans person, task, and organization, reflecting its role as a cross-cutting behavioral driver. This mapping was constructed after implementation to aid interpretation and was not used prospectively to design the bundle.

Aims

The prespecified primary outcome was the change in composite perceived ICU calmness among nursing staff, assessed using a retrospective paired then-now design. Because the pre-intervention rating was obtained retrospectively after exposure to the intervention had begun, this outcome is interpreted as an observed association rather than definitive evidence of a causal effect. Secondary and exploratory/descriptive outcomes included staff work environment perceptions (handover quality, handover confidence, freedom from noise-related work disruption, freedom from work-related stress, job satisfaction, and freedom from workflow disturbance); post-intervention perceived workload, assessed descriptively using an adapted NASA Task Load Index (NASA-TLX) instrument; and patient/surrogate perceptions of ICU environmental reassurance, assessed descriptively and cross-sectionally post-intervention only.

## Materials and methods

Study design

This was a single-center, uncontrolled, before-after quality improvement study employing a retrospective paired then-now assessment design for primary nursing staff outcomes. For each nurse, the retrospective pre-intervention ("then") rating and the post-intervention ("now") rating were collected using the same paired instrument administered on two occasions about 10 days apart. Because the pre-intervention rating was itself collected retrospectively after intervention exposure had begun, rather than as a true prospective baseline recorded before any exposure, this approach retains some protection against response-shift bias relative to conventional pre-post surveys; however, since the two ratings were not obtained in a single encounter, this protection is partial, and the design remains susceptible to recall drift between the two administrations in addition to retrospective recall bias. The study follows Standards for Quality Improvement Reporting Excellence (SQUIRE) guidelines [14]. Secondary staff outcomes were assessed using the same paired instrument. Post-intervention perceived workload was assessed cross-sectionally using an adapted NASA-TLX instrument [15]. Patient/surrogate perception of the ICU environment was assessed cross-sectionally post-implementation.

Study setting and context

This study was conducted in the adult intensive care unit of GEM Hospital, Coimbatore, Tamil Nadu, India, a tertiary care center with subspecialty expertise in gastrointestinal surgery, hepatobiliary and pancreatic surgery, liver transplantation, kidney transplantation, and adult oncology. The ICU is a 20-bed mixed medical-surgical unit staffed by consultant intensivists, critical care nurses, ICU technicians, and a clinical pharmacologist embedded within the ICU team. The study was conducted across mixed eight- and 12-hour shifts with variable nurse-to-patient ratios reflecting the mixed medical-surgical case mix. The principal investigator and charge nurses jointly led implementation, sharing responsibility for daily reinforcement and behavioral modeling. No major organizational disruptions, staffing crises, or leadership changes occurred during the study period that would independently influence the measured outcomes.

Prior to the intervention, the unit operated without a formalized noise reduction or behavioral calmness protocol. Informal observations by the principal investigator identified recurrent patterns of high-volume communication during routine care, non-individualized alarm limits, absence of structured handover processes, and limited provision for staff emotional recovery during shifts. These contextual factors prompted the development of the CALMER bundle.

CALMER bundle: intervention description

Prior to implementing the CALMER bundle, routine ICU practice included standard communication and workflow processes, along with usual patient-specific adjustments to alarm settings as clinically indicated. No formalized noise reduction protocol, structured handover framework, or behavioral calmness initiative was in place.

Communication Discipline

Communication discipline targeted the quality, structure, and tone of all verbal interactions within the ICU. Key behaviors included delivering structured shift handovers using the ISBAR (Identify-Situation-Background-Assessment-Recommendation) framework; avoiding staff clustering at the nurses' station; minimizing avoidable interruptions during handovers; maintaining a controlled tone across the ICU; and providing visitors with structured guidance on noise limitation and calm communication.

Alarm Discipline

The alarm discipline component aimed to enhance alarm relevance and reduce non-actionable alarm burden through nurse-led review and configuration. Bedside nurses, in collaboration with the attending intensivist, reviewed and individualized alarm parameters for their assigned patients during daily clinical rounds, adjusting thresholds according to the patient's evolving clinical condition while ensuring that critical physiological alerts remained appropriately sensitive. Alarm acknowledgment and clinical response remained a nursing responsibility at all times, consistent with standard ICU practice.

Leadership Modeling

Leadership modeling formalized senior clinical staff as visible behavioral exemplars. Specific behaviors included senior staff demonstrating calm, structured communication; maintaining an active leadership presence during high-risk periods; and pre-assigning emergency roles at shift commencement. At each shift commencement, the charge nurse designated primary and secondary leads for three defined emergency roles: intubation lead, drug lead, and crash cart/monitor in charge. This dual-designation system eliminated role ambiguity during emergencies.

Mindful Workflow and Equipment Handling

Mindful workflow and equipment handling addressed noise generated through physical movement and equipment use within the ICU. Key behaviors included quiet, deliberate handling of monitors, trolleys, ventilators, and other clinical equipment, and avoiding bedside crowding during routine care activities.

Emotional Reset

Emotional reset provided structured opportunities for emotional decompression throughout the shift. Nursing staff could approach their team leader at any point during the shift to discuss work-related stress. The unit designated a dedicated quiet space to support these intra-shift discussions.

Reinforcement

Reinforcement was designed to support sustainability through daily visual reminders, peer accountability structures, and positive reinforcement. Where staff demonstrated inconsistent adherence, the unit engaged them as CALMER Champions through supportive coaching rather than corrective action, promoting shared ownership of behavioral standards.

Figure 1 summarizes the CALMER intervention framework, including the six behavioral domains and their component practices.

**Figure 1:**
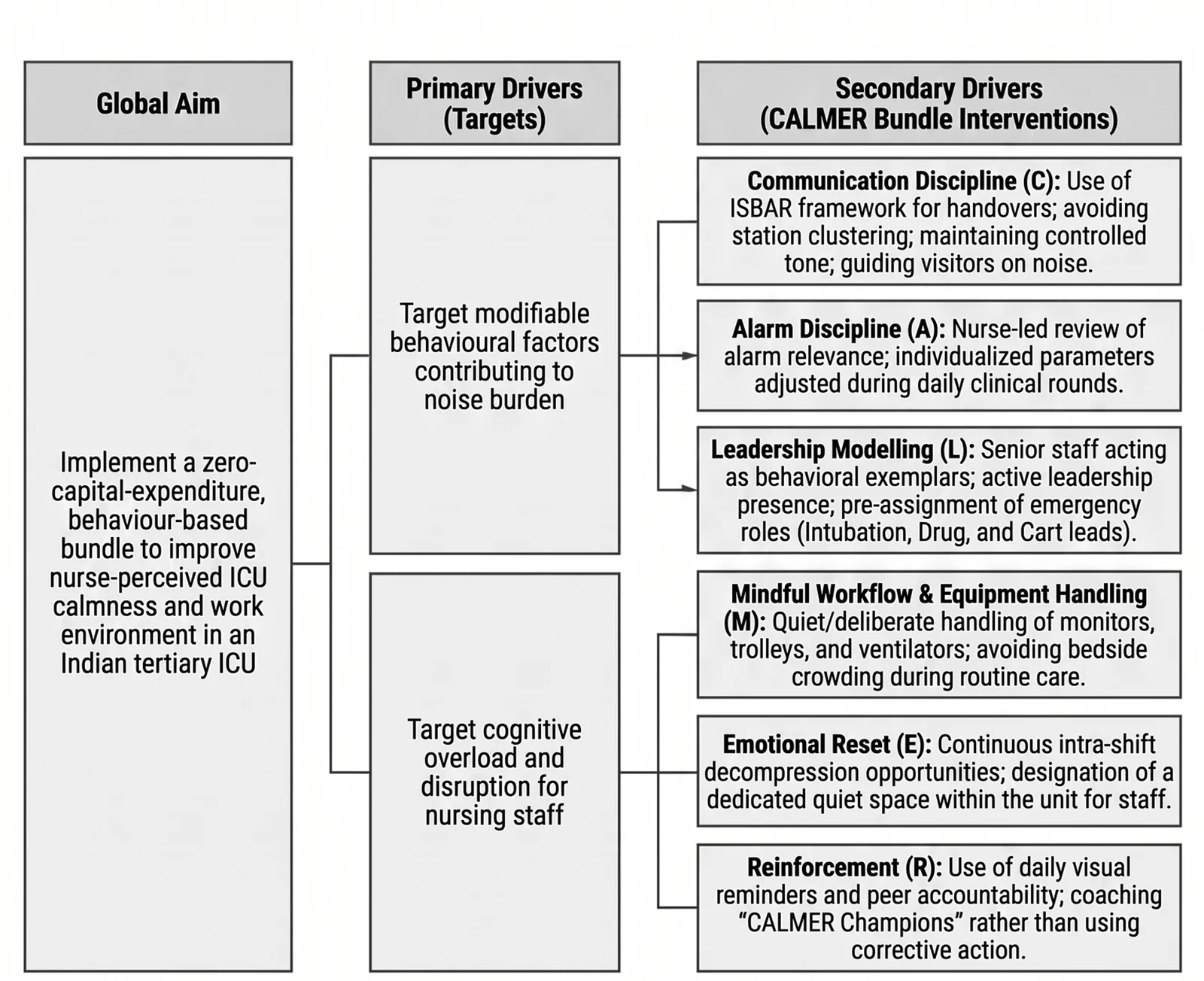
CALMER bundle driver diagram ICU: intensive care unit

Implementation strategy

Months 1-3 comprised staff engagement, CALMER orientation (100% completion), safe-to-fail rollout, and active implementation with leadership reinforcement. Month 4 was the nurse data collection phase, during which the principal investigator personally administered the pre-intervention ("then") questionnaire to each nurse individually; the post-intervention ("now") questionnaire was administered to the same nurse about 10 days later, after continued implementation exposure. Months 5-6 focused on consolidation, sustainability auditing, and patient/surrogate data collection.

Two implementation principles underpinned staff adoption. The staff-first ‘What’s in it for me?’ (WIIFM) approach positioned the bundle primarily as a tool to reduce nursing cognitive load and workflow friction and was intended to encourage staff buy-in before emphasizing patient benefits. The safe-to-fail principle explicitly reassured staff that all changes could be reversed if found ineffective and was intended to reduce baseline resistance and encourage staff ownership of the cultural shift; these effects on adoption resistance and ownership were not directly measured.

All nursing staff received CALMER orientation prior to data collection. Initial staff orientation required less than 30 minutes per nurse; reinforcement meetings were embedded within existing shift handover structures and varied in duration. Visual reminders reinforcing bundle components were displayed at nursing stations and bedside workspaces throughout the intervention period. No additional infrastructure expenditure was incurred. The bundle was implemented as planned without major protocol deviation; no components were suspended or modified during the study period.

Data collection timeline

The principal investigator personally administered nursing staff questionnaires to each nurse during month 4 of the study period. For each nurse, the retrospective pre-intervention ("then") and post-intervention ("now") ratings were collected using the same instrument administered on two occasions approximately 10 days apart, yielding a paired then-now assessment design. Prospective baseline data collection was not feasible given the study's quality improvement-first origin. Because the pre-intervention rating was itself collected retrospectively after intervention exposure had begun, rather than as a true prospective baseline, this design retains some protection against response-shift bias relative to conventional pre-post surveys; however, since the two ratings were obtained on separate occasions rather than in a single encounter, this protection is only partial. The then-now approach increases susceptibility to recall bias, as pre-intervention ratings rely on retrospective memory of conditions approximately four months prior, and the approximately 10-day interval between the paired ratings introduces further potential for recall drift between the two administrations; this limitation is acknowledged and discussed in the Limitations section. Patient/surrogate perception questionnaires were administered cross-sectionally during months 5 and 6 of the study period, at discharge or during a stable phase of the ICU admission.

Outcome measures

Primary Outcome

The primary outcome was the difference in composite perceived ICU calmness score between recall-based pre-intervention and post-intervention assessments among nursing staff. The composite score was derived from eleven items across four domains assessed on a five-point Likert scale (1 = strongly disagree, 5 = strongly agree): environmental calmness (Q1, Q2, Q8, Q9), cognitive focus (Q3, Q4, Q5), communication quality (Q6, Q7), and workflow coordination (Q10, Q11). Three items were negatively worded: Q2 (shouting frequency in ICU), Q3 (noise-related concentration difficulty), and Q10 (bedside crowding during routine care), and were reverse-scored prior to analysis using the formula: score = 6 − raw score on a 1-5 scale. Following reversal, higher scores consistently indicate greater perceived calmness across all 11 items.

Secondary Outcome

Work environment: Six items assessing handover quality, handover confidence, freedom from noise-related work disruption, freedom from work-related stress, job satisfaction, and freedom from workflow disturbance (Q12-17), reported as a composite staff work environment score, assessed in the same 28 eligible bedside nurses as the primary outcome. Q14 and Q15 were reverse-scored prior to analysis; higher scores indicate better outcomes throughout.

Workload (NASA-TLX): Post-intervention perceived workload was assessed using an adapted NASA-TLX instrument across six domains, rated on a 1-5 scale. Unlike the calmness and work environment outcomes, this instrument was administered to all 40 nursing staff, including the 12 team leaders and nursing assistants excluded from the primary and work environment analyses, as perceived workload is not specific to direct bedside patient assignment and applies across all nursing roles in the unit.

Patient/surrogate perception: Post-intervention perception of ICU environmental reassurance was assessed cross-sectionally across five domains in 100 patients or their surrogates. All questionnaires were administered anonymously.

Implementation fidelity audit

As part of ongoing sustainment monitoring following the primary data collection period, a structured compliance audit tool comprising 15 behaviorally anchored items (scored 0, 0.5, or 1 per item) mapped to the six CALMER domains was administered across nursing shifts. This represents a later-phase implementation-fidelity assessment rather than continuous audit coverage across the full study period: audits were conducted between May 7 and June 30, 2026. Domain-wise compliance was expressed as the percentage of the maximum achievable score across all scored items within each domain.

Questionnaire development and validation

This evaluation used four study instruments: three were developed specifically for this study, while the workload instrument was locally adapted from the established NASA-TLX framework. All instruments underwent partial validation only: internal consistency was assessed, but formal psychometric validation, including independent review, pilot testing, and factor analysis, was not performed before administration. The instruments comprised (i) an 11-item ICU calmness questionnaire (Cronbach's alpha = 0.831); (ii) a six-item staff work environment questionnaire; (iii) a locally adapted workload perception scale informed by the NASA-TLX framework, rated on a 1-5 scale, with findings not directly comparable with published NASA-TLX normative data; and (iv) a five-item patient/surrogate perception questionnaire.

All instruments were constructed using a deductive item-to-domain mapping approach, with items systematically derived from the behavioral objectives of each CALMER bundle component. Internal consistency of the primary calmness instrument was assessed using Cronbach's alpha (threshold ≥0.70). Item-level reliability was evaluated through corrected item-total correlation analysis (threshold r > 0.30 for item retention). The Appendices provide the full item wording for all four instruments.

Questionnaires were administered in English; when respondents requested clarification of an item's meaning, the principal investigator provided verbal clarification in Tamil.

Statistical analysis

Descriptive statistics summarized participant and setting characteristics. Continuous data were reported as medians and interquartile ranges (IQRs). Each nurse's primary composite score was calculated as the median of their 11 individual item scores (Q1-Q11); the group result was reported as the median and IQR of individual composite scores across eligible nurses. Paired ordinal pre-post data were analyzed using the Wilcoxon signed-rank test. Effect size was calculated as r = z/√n, with 95% confidence intervals (CIs) derived using Fisher's z-transformation. Internal consistency was assessed using Cronbach's alpha. Analyses were performed using Python version 3.12 (Python Software Foundation, Beaverton, OR, USA) with the SciPy statistical library (scipy.stats.wilcoxon; normal approximation, no continuity correction; SciPy Project (NumFOCUS, Inc.), Austin, TX, USA). Effect size r and 95% CIs were computed as described above. A two-tailed p-value of <0.05 was considered statistically significant. These statistical analyses, including the Wilcoxon signed-rank tests, effect sizes, and CIs, were executed by a generative AI tool operating within the Python SciPy environment described above, at the authors' direction and applied to the study's raw response data; all statistical outputs were independently reviewed and verified by the authors, who take full responsibility for their scientific accuracy and integrity. Additionally, the authors conducted a contextual literature search to support the Introduction and Discussion in July 2026 using PubMed, supplemented by the AI-assisted research platforms Consensus (Consensus, San Francisco, CA, USA) and Claude (Deep Research; Anthropic, San Francisco, CA, USA); the authors manually verified retrieved references against source publications before citation.

Ethical considerations and study registration

The CALMER bundle was initiated as a unit-level quality improvement program in January 2026. The January-March 2026 period constituted operational implementation and staff orientation only; no study outcome data were collected during this phase. The Institutional Ethics Committee of Kongunad Hospitals Private Limited approved the study before formal data collection began (approval number: IEC/KH/2026/03/2603; approval date: March 26, 2026). The study title was subsequently revised; the institution formally documented this revision after study completion (approval number: KNH/ACADEMIC/04032025, approval date: August 15, 2026). Individual written informed consent was waived, as the study was classified as a quality improvement evaluation. Participation in all questionnaires (nursing staff, patient, and surrogate) was voluntary and anonymous. The study was not prospectively registered on the Clinical Trials Registry of India (CTRI), as it was initiated as a unit-level quality improvement program. Findings are reported in accordance with SQUIRE 2.0 guidelines [14].

Participants

Nursing Staff

Inclusion criteria: Registered ICU nursing staff (nurses, team leaders, and nursing assistants) with ≥1 month of continuous ICU work experience, actively working in the unit prior to and during the intervention period, and willing to complete both recall-based and post-intervention questionnaires.

Analysis population for paired calmness assessment: The primary calmness and secondary work environment questionnaires were administered to all 40 nursing staff. Twelve nurse team leaders and nursing assistants without individual patient assignment left specific items blank because they did not apply to their roles, principally items assessing bedside noise exposure and handovers received, and, in two cases, an additional work environment item, because these items assessed perceptions directly related to bedside patient-care responsibilities not shared by staff without individual patient assignments. Complete-case analysis therefore included the 28 nurses with fully complete paired responses across all primary (Q1-11) and secondary (Q12-17) items (28/40, 70% completion). Nurses with <1 month of ICU experience, those who joined after CALMER implementation, temporary or rotational staff, and those on prolonged leave were not part of the 40-person roster assessed for participation. All 40 nursing staff, including the 12 with incomplete calmness/work environment responses, completed the post-intervention NASA-TLX workload assessment, as perceived workload is not specific to direct patient assignment and applies across all nursing roles in the unit.

Patients/Surrogates

Inclusion criteria: Patients with ICU stays exceeding 24 hours who were clinically stable and conscious, or their available surrogates, willing to participate.

Exclusion criteria: ICU stay <24 hours, deep sedation, active delirium, mechanical ventilation, hemodynamic instability, or refusal to participate.

Figure 2 summarizes the participant flow, including data collection and analysis population branching.

**Figure 2:**
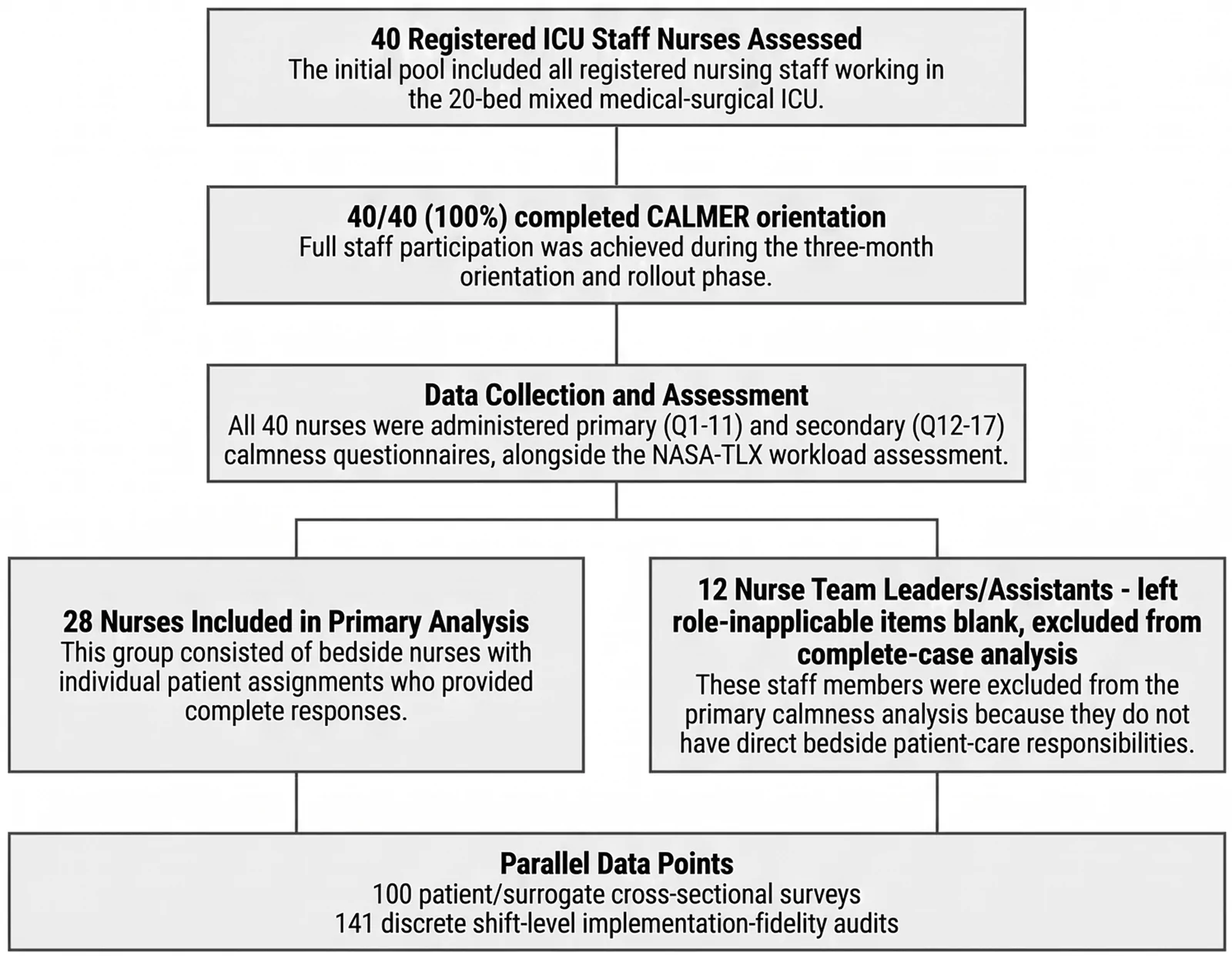
Study flow diagram ICU: intensive care unit, NASA-TLX: NASA Task Load Index

## Results

Participant flow and study population

Of the 40 registered ICU nursing staff assessed for participation, all 40 completed CALMER orientation (100% orientation rate) and were administered the primary (Q1-11) and secondary (Q12-17) calmness and work environment questionnaires. Twenty-eight nurses with individual patient assignments provided fully complete paired pre- and post-intervention responses across all items. The remaining 12 (nurse team leaders and nursing assistants without individual patient assignment) left role-inapplicable items blank (principally Q3 and Q5 and Q13 for two respondents), consistent with these items assessing perceptions tied to direct bedside patient-care responsibilities not performed by this group; complete-case analysis was therefore restricted to the 28 nurses with fully complete responses (28/40, 70% completion). All 40 staff completed the post-intervention NASA-TLX workload assessment. One hundred patients or their surrogates completed the post-intervention cross-sectional environmental perception questionnaire.

Table 1 summarizes study characteristics and the analysis populations.

**Table 1:** Baseline characteristics and study population SQUIRE 2.0: Standards for Quality Improvement Reporting Excellence, ICU: intensive care unit

Variable	Value
Study design	Single-center, uncontrolled before-after quality improvement study; retrospective paired then-now assessment design
Study duration	6 months (January-June 2026)
Setting	20-bed mixed medical-surgical ICU, GEM Hospital, Coimbatore
Total nursing staff assessed	40
CALMER orientation completion	40/40 (100%)
Nurses with complete paired calmness responses (analysis population)	28/40 (70%); bedside nurses with individual patient assignment
Staff with incomplete responses (role-inapplicable items left blank)	12 (nurse team leaders and nursing assistants without individual patient assignment)
Patient/surrogate respondents	100 (cross-sectional, post-intervention)
Questionnaire internal consistency (Cronbach's α)	0.831 (11-item primary calmness tool)
Reporting framework	SQUIRE 2.0

Instrument reliability

The 11-item composite ICU calmness tool showed good internal consistency (Cronbach's alpha = 0.831) among the 28 eligible bedside nurses. All corrected item-total correlations exceeded the accepted threshold of r > 0.30, supporting retention of all items in the composite score.

Primary outcome

Composite ICU Calmness Score

Among the 28 nurses with complete paired responses, the composite ICU calmness score improved significantly following CALMER implementation. The pre-intervention median score was 2.0 (IQR 1.75-2.0) compared to a post-intervention median of 4.0 (IQR 4.0-4.0), representing a statistically significant improvement with a large effect size (Wilcoxon signed-rank test, p < 0.001, r = 0.910, 95% CI 0.813-0.958).

All four calmness domains demonstrated statistically significant improvements (all p < 0.001) with large effect sizes. Environmental calmness improved from a median of 1.5 (IQR 1.0-2.0) to 4.0 (IQR 4.0-4.0) (r = 0.874, 95% CI 0.743-0.940). Cognitive focus improved from 2.0 (IQR 2.0-3.0) to 4.0 (IQR 4.0-5.0) (r = 0.858, 95% CI 0.713-0.933). Communication quality improved from 1.75 (IQR 1.5-2.5) to 4.0 (IQR 4.0-4.5) (r = 0.842, 95% CI 0.684-0.925). Workflow coordination improved from 1.75 (IQR 1.5-3.0) to 3.75 (IQR 3.5-4.0) (r = 0.874, 95% CI 0.743-0.940). Table 2 presents the full results.

**Table 2:** Primary outcome: composite and domain-wise ICU calmness scores (n = 28) All scores are based on a 1-5 Likert scale. Higher scores indicate greater perceived calmness. † Q2 (shouting frequency), Q3 (noise-related concentration difficulty), and Q10 (bedside crowding) were reverse-scored before analysis (score = 6 − raw score); thus, higher scores consistently indicate greater perceived calmness across all items. Wilcoxon signed-rank test. Effect size r = z/√n; 95% CIs were calculated using Fisher’s z-transformation. Domain analyses are exploratory; the primary prespecified outcome is the composite score, and no correction for multiple comparisons was applied to domain-level results. The investigators prespecified the ≥2-point improvement threshold for responder classification a priori. IQR: interquartile range, CI: confidence interval, ICU: intensive care unit

Domain	Pre-CALMER median (IQR)	Post-CALMER median (IQR)	Effect size r (95% CI)	p-value
Composite calmness score (Q1-11)	2.0 (1.75-2.0)	4.0 (4.0-4.0)	0.910 (0.813-0.958)	<0.001
Environmental calmness (Q1, Q2, Q8, Q9)	1.5 (1.0-2.0)	4.0 (4.0-4.0)	0.874 (0.743-0.940)	<0.001
Cognitive focus (Q3, Q4, Q5)	2.0 (2.0-3.0)	4.0 (4.0-5.0)	0.858 (0.713-0.933)	<0.001
Communication quality (Q6, Q7)	1.75 (1.5-2.5)	4.0 (4.0-4.5)	0.842 (0.684-0.925)	<0.001
Workflow coordination (Q10, Q11)	1.75 (1.5-3.0)	3.75 (3.5-4.0)	0.874 (0.743-0.940)	<0.001

Responder Distribution

Of the 28 nurses in the primary analysis, 26 (92.9%) improved by ≥2 points in composite calmness score. The investigators prespecified this ≥2-point threshold, and it is not derived from an externally validated minimal clinically important difference for this instrument. The remaining two nurses (7.1%) demonstrated a 1-point improvement. No nurse reported no change or a decline in calmness score post-intervention. All 28 nurses improved by at least 1 point (100% any-improvement rate).

Figure 3 shows individual-level change in composite calmness score for each of the 28 nurses.

**Figure 3:**
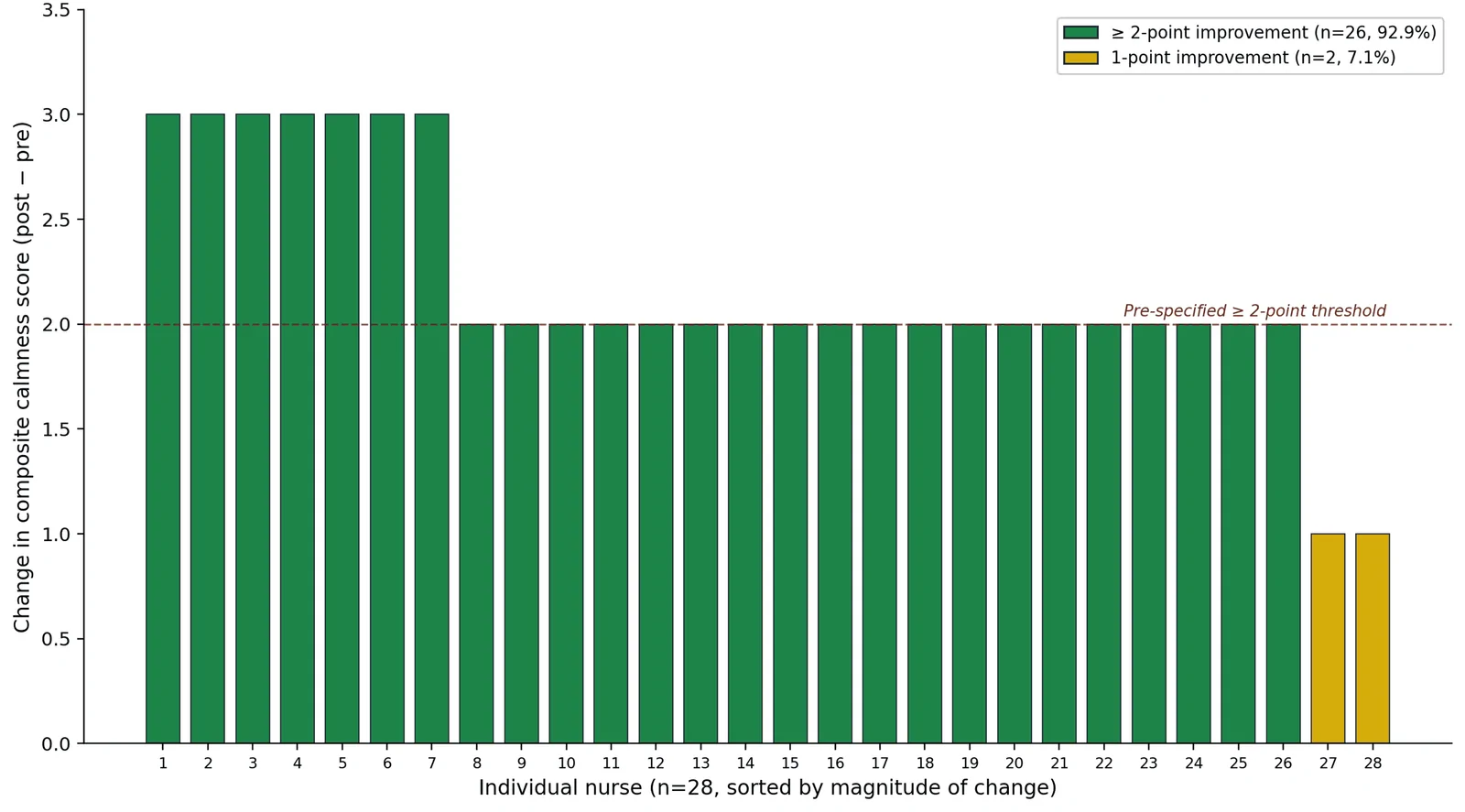
Waterfall plot showing the individual nurse composite calmness score change (n = 28)

Secondary outcome

Staff Work Environment and Perception

The composite staff work environment score, derived from six items (Q12-17) assessing handover quality, handover confidence, freedom from noise-related work disruption, freedom from work-related stress, job satisfaction, and freedom from workflow disturbance, improved significantly following CALMER implementation. The pre-intervention composite median was 2.25 (IQR 2.0-3.0) compared to a post-intervention median of 4.0 (IQR 4.0-4.0) (Wilcoxon signed-rank test, p < 0.001, effect size r = 0.874, 95% CI 0.743-0.940). Wilcoxon signed-rank tests were also applied at the individual item level (Q12-17); all six items improved significantly (all p < 0.001), with effect sizes ranging from r = 0.751 to r = 0.872 (Table 3). These item-level analyses are exploratory and uncorrected for multiple comparisons. Notably, Q16 (job satisfaction) improved significantly from a pre-intervention median of 3.5 (IQR 2.0-4.0) to 4.0 (IQR 4.0-5.0) (r = 0.763, p < 0.001), consistent with the overall pattern of improvement in staff work experience.

**Table 3:** Secondary outcome: composite and item-wise staff work environment scores (n = 28) All scores are based on a 1-5 Likert scale. Higher scores indicate a better overall perceived work environment. The Wilcoxon signed-rank test was applied to the composite score and to each individual item; item-level tests are exploratory, and no correction for multiple comparisons was applied. † Q14 and Q15 were reverse-scored before analysis; higher scores indicate greater freedom from noise-related work disruption and work-related stress, respectively. Effect size r = z/√n; 95% CIs were calculated using Fisher’s z-transformation. All n = 28 (bedside nurses with complete Q1-Q17 responses). IQR: interquartile range, CIs: confidence intervals

Outcome/item	Pre-CALMER median (IQR)	Post-CALMER median (IQR)	Effect size r (95% CI)	p-value
Composite staff work environment score (Q12-17)	2.25 (2.0-3.0)	4.0 (4.0-4.0)	0.874 (0.743-0.940)	<0.001
Handover quality (Q12)	2.0 (2.0-3.0)	4.0 (4.0-4.0)	0.872 (0.739-0.939)	<0.001
Handover confidence (Q13)	3.0 (2.0-4.0)	4.0 (4.0-4.2)	0.751 (0.525-0.878)	<0.001
Freedom from noise disruption (Q14)†	2.0 (2.0-3.0)	4.0 (4.0-4.0)	0.800 (0.609-0.903)	<0.001
Freedom from work-related stress (Q15)†	2.0 (2.0-3.0)	4.0 (3.8-4.0)	0.751 (0.525-0.878)	<0.001
Job satisfaction (Q16)	3.5 (2.0-4.0)	4.0 (4.0-5.0)	0.763 (0.545-0.884)	<0.001
Freedom from workflow disturbance (Q17)	2.0 (2.0-3.0)	4.0 (4.0-4.0)	0.859 (0.715-0.933)	<0.001

Post-Intervention Workload: NASA-TLX

Post-intervention workload was assessed in all 40 nurses using an adapted NASA-TLX instrument rated on a 1-5 scale. The overall workload median was 3.0 (IQR 2.4-4.0), the scale midpoint, measured post-implementation. The investigators describe this descriptively as a midpoint workload rating, as no externally validated severity bands exist for this adapted 1-5 format. Temporal demand was the highest-rated subdomain (median 4.0, IQR 3.0-4.0), while frustration was the lowest-rated demand subdomain (median 2.0, IQR 2.0-4.0). This scale-midpoint workload rating was observed alongside significant improvements in perceived calmness; however, the absence of a pre-intervention workload measure precludes assessment of changes in workload (Table 4).

**Table 4:** Post-intervention NASA-TLX workload profile (n = 40) NASA-TLX was assessed post-intervention only (n = 40; all nursing staff, including team leaders and nursing assistants). An adapted 1-5 scale was used (1 = lowest load, 5 = highest load). † The Performance domain is presented as a reversed load score; the original item, “I am satisfied with my performance,” had a median of 4.0 (IQR, 3.0-4.0), indicating favorable self-rated performance. NASA-TLX: NASA Task Load Index, IQR: interquartile range

NASA-TLX domain	Post-CALMER median (IQR)
Mental demand	3.0 (2.0-4.0)
Physical demand	3.5 (2.0-4.0)
Temporal demand	4.0 (3.0-4.0)
Effort	3.0 (2.8-4.0)
Frustration	2.0 (2.0-4.0)
Performance†	2.0 (2.0-3.0)
Overall workload (TLX median)	3.0 (2.4-4.0) - scale midpoint

Patient/Surrogate Perception of ICU Environment

One hundred patients or their surrogates completed the post-intervention cross-sectional environmental perception questionnaire. Perceptions were uniformly high across all five assessed domains, with a median score of 5.0 (IQR 5.0-5.0) for each domain. Staff responsiveness received the highest endorsement (99% rated 5). Clear and reassuring communication and confidence in care were each rated 5 by 95% of respondents. The lowest, but still highly favorable, rating was for a calm and organized ICU environment, with 81% rating it 5 and 19% rating it 4, and no respondent rating it below 4. As this was a cross-sectional post-intervention assessment without a pre-intervention comparator, these findings are reported descriptively and should not be interpreted as demonstrating improvement from a baseline (Table 5).

**Table 5:** Patient/surrogate post-intervention environmental perception (n = 100) Cross-sectional post-intervention assessment (n = 100). Scores are based on a 1-5 Likert scale (1 = strongly disagree, 5 = strongly agree). IQR = interquartile range. One respondent (1%) rated confidence in care received as 3; the remaining 99% rated it 4 or 5. One respondent (1%) rated staff responsiveness as 3; the remaining 99% rated it 4 or 5. ICU: intensive care unit, IQR: interquartile range

Domain	Median (IQR)	Score 5 (%)	Score 4 (%)	Range
Calm and organized ICU environment	5.0 (5.0-5.0)	81%	19%	4-5
Clear and reassuring communication	5.0 (5.0-5.0)	95%	5%	4-5
Confidence in care received	5.0 (5.0-5.0)	95%	4%	3-5
Staff responsiveness	5.0 (5.0-5.0)	99%	0%	3-5
Overall ICU experience	5.0 (5.0-5.0)	94%	6%	4-5

Safety

Routine local safety records identified no alarm-related sentinel events or intervention-associated adverse incidents during the study period.

Implementation fidelity

A total of 141 discrete shift-level implementation-fidelity audits were conducted during the audit window described in the Materials and Methods. Table 6 presents domain-level compliance. Overall compliance across all domains was 82.9%. Leadership modeling showed the highest domain-level compliance (91.8%), while emotional reset (76.2%) and mindful workflow and equipment handling (77.2%) were comparatively lower, making them priority areas for ongoing reinforcement.

**Table 6:** Implementation fidelity: CALMER compliance audit by domain (May 7 to June 30, 2026, n = 141 audits) Compliance (%) = (sum of item scores achieved ÷ maximum achievable score) × 100, summed across all audits within the period for items mapped to each domain. Three entries recorded as the nonnumeric character “O” (handover calmness item) were treated as 0. Blank or not-applicable entries (n = 6 across five items) were excluded from the denominator for the corresponding item. Domain item counts were as follows: communication discipline (four items: handover calmness, nurses’ station calmness, nonclinical discussion redirection, and calm communication), alarm discipline (three items), leadership modeling (two items), mindful workflow and equipment handling (two items), emotional reset (two items), and reinforcement (two items).

CALMER domain	Compliance (%)	Items scored (n)
Overall	82.9%	2109
Communication discipline	82.9%	563
Alarm discipline	87.9%	422
Leadership modeling	91.8%	282
Mindful workflow and equipment handling	77.2%	279
Emotional reset	76.2%	282
Reinforcement	78.5%	281

## Discussion

Summary of key findings

This quality improvement study evaluated the feasibility and perceived impact of the CALMER bundle in a 20-bed tertiary mixed medical-surgical ICU. The principal finding was a statistically significant, large-magnitude improvement in the composite ICU calmness score after implementation, with the median rising from 2.0 to 4.0 on a five-point Likert scale (effect size r = 0.910, 95% CI 0.813-0.958, p < 0.001). All four calmness domains improved uniformly, and the composite staff work environment score moved in parallel (r = 0.874, 95% CI 0.743-0.940, p < 0.001). Perceived workload, assessed post-intervention with an adapted NASA-TLX instrument, had a median of 3.0/5 (the scale midpoint), alongside the calmness gains. All 28 nurses improved, and 92.9% reached the pre-specified ≥2-point responder threshold. Later-phase implementation-fidelity auditing (141 shift-level audits, May 7 to June 30, 2026) showed overall compliance of 82.9%, highest for leadership modeling (91.8%) and comparatively lower for emotional reset (76.2%) and mindful workflow and equipment handling (77.2%).

Contextualization within existing literature

The CALMER bundle builds on and extends the existing evidence base for reducing ICU noise and cognitive burden. Tronstad et al. reported measurable improvements in sound levels and patient comfort through a structured sound reduction bundle [2]. At the same time, Darbyshire and Young confirmed that mapping noise to modifiable behavioral sources is a reproducible methodology [16]. Konkani et al. established the feasibility of behavior-based ICU noise reduction in resource-realistic settings [1]. A concurrently registered Indian trial (CTRI/2025/10/096371) targets delirium prevention in mechanically ventilated patients through a multimodal, patient-centered sensory and reorientation bundle, of which noise control is one component; its primary outcome is delirium/agitation incidence rather than staff-perceived calmness. This differs fundamentally from CALMER, where staff behavioral culture change, not patient-level sensory intervention, is both the intervention and the measured construct. CALMER also departs from the prevailing paradigm in ICU noise research, which is overwhelmingly anchored on objective decibel measurement, by adopting nurse-rated perceived calmness as its primary outcome. This choice is supported by evidence that objective sound-level measurements may not fully capture the behavioral and experiential dimensions targeted by multicomponent ICU noise interventions. Comparable bundles have produced statistically significant but clinically small changes in sound levels despite meaningful improvements in alarm burden [2,17], distractions, and staff and patient experience [17]. While prior quality-improvement work outside the ICU has used patient-perceived quietness (e.g., HCAHPS "always quiet" scores) as a primary endpoint, using nurse self-rated perceived calmness as the primary outcome of a zero-capital-expenditure, purely behavioral bundle in an adult ICU is, to our knowledge, novel.

Three features distinguish CALMER from previously published interventions. First, the alarm discipline component emphasizes nurse-led, individualized alarm configuration reviewed during daily clinical rounds rather than reliance on default thresholds, keeping all alarm-related clinical judgment within the nursing and medical team. Second, the emotional reset component provides a structured opportunity for emotional decompression facilitated by team leaders during the shift. Third, the reinforcement component reframes inconsistent adherence as a leadership development opportunity through CALMER champion engagement, based on supportive coaching rather than corrective action.

The SEIPS framework offers a useful lens for contextualizing CALMER's multi-level approach: addressing person, task, environment, and organizational factors simultaneously [6]. Pronovost's framework supports bundled interventions, with leadership engagement as an essential driver of implementation [9]. Emerging evidence further suggests that behavioral change in ICU noise reduction is most sustainable when leadership drives it, and senior clinical staff model it, consistent with CALMER's leadership modeling domain [9].

Calmness-workload relationship: a hypothesis-generating observation

The observation that calmness and workload may appear partially dissociable warrants cautious interpretation. Despite a post-intervention NASA-TLX median of 3.0/5 (the scale midpoint), which the investigators interpreted descriptively as a midpoint workload rating, all 28 nurses demonstrated improved calmness. Importantly, the absence of a pre-intervention workload measure means that any change in workload perception attributable to CALMER cannot be formally assessed; the NASA-TLX profile reflects a single post-intervention time point only. This pattern generates the hypothesis that perceived ICU calmness may be influenced by behavioral and environmental structure independent of workload quantity. However, this requires prospective confirmation and should not be interpreted as established.

Patient/surrogate perception

Patient/surrogate perceptions of the ICU environment, assessed cross-sectionally during months 5 and 6, were strongly positive across all five assessed domains. As this was a cross-sectional post-intervention assessment without a pre-intervention comparator, these findings are reported descriptively. They should not be interpreted as demonstrating improvement from a baseline, nor as evidence of a causal link to the CALMER bundle specifically. High satisfaction scores recorded at or near ICU discharge may partly reflect survivorship and gratitude biases, well-recognized phenomena in critical care satisfaction surveys, in which relief at surviving critical illness and baseline appreciation for nursing care can inflate ratings independent of any specific intervention. Accordingly, these data are presented as a secondary, hypothesis-generating measure of post-intervention environmental acceptability rather than as confirmatory evidence of the bundle's impact on patient experience.

Limitations

This study has several limitations. First, the retrospective paired then-now design is inherently susceptible to recall bias, as participants retrospectively rated their pre-intervention perceptions while completing post-intervention assessments [14,18,19]. The then-now approach was chosen specifically to mitigate response-shift bias, a recognized limitation of traditional pre-post designs, representing a deliberate methodological trade-off.

Second, the study was conducted at a single center, limiting generalizability. Third, all primary and secondary outcomes are perception-based, self-reported measures using investigator-developed instruments that did not undergo formal multi-expert panel review or independent pilot testing prior to administration. Unlike validated instruments used in comparable multicenter ICU noise-perception work [20], the CALMER questionnaires have not undergone formal psychometric validation beyond internal consistency testing. Fourth, the study assessed outcomes using instruments aligned with the same intervention domains and designed by the same principal investigator, increasing susceptibility to common-method bias.

Fifth, the study did not include objective acoustic measurements. Sixth, the study used an adapted NASA-TLX instrument rated on a 1-5 scale rather than the original validated 0-100 format, precluding direct comparison with published NASA-TLX normative data [15].

Seventh, all 40 nursing staff were administered the primary and secondary questionnaires; complete-case analysis was restricted to the 28 nurses with fully complete paired responses (28/40, 70% completion), as the remaining 12 team leaders/nursing assistants left role-inapplicable items blank rather than declining to participate, reflecting item-level non-applicability rather than attrition or non-response. Face-to-face administration by the principal investigator increases susceptibility to social desirability bias despite the anonymous data-collection format.

Eighth, the study is subject to the Hawthorne effect: nursing staff knew their unit was the subject of a visible, principal-investigator-led improvement initiative, and this awareness may have shaped both behavior and self-reported perceptions, regardless of genuine environmental change. This risk is compounded by the face-to-face administration of the questionnaire by the principal investigator, who also designed and implemented the intervention; despite the anonymous format, direct administration by an authority figure invested in the bundle's success plausibly increased susceptibility to social desirability and acquiescence bias and may have contributed to the uniformly positive response pattern (100% of nurses improving, no decliners). This combination of biases should be weighted heavily when interpreting the magnitude, though not necessarily the direction, of the observed effects. The magnitude and consistency of this uniformity warrant explicit emphasis: composite and domain-level effect sizes exceeded r = 0.84 across every measured domain, and not a single nurse among the 28 in the primary analysis reported a decline. This uniform pattern is atypical for a naturally occurring behavioral or perceptual shift. It is more consistent with response bias operating alongside, or in place of, genuine change than with an effect of this size occurring in isolation. Readers should interpret the reported effect sizes as an upper-bound estimate of CALMER's true effect pending independent, blinded, or third-party-administered replication; the planned multicenter validation (see Future Directions) is intended to address this by removing principal-investigator administration from the data-collection pathway.

Ninth, the study was not prospectively registered on the CTRI, reflecting its quality improvement-first origin. Tenth, the large observed effect sizes (r = 0.910) may reflect a combination of genuine environmental change, response-shift effects, Hawthorne and social desirability effects, and increased staff awareness rather than representing the isolated effect of the CALMER bundle alone.

Eleventh, the 12 nurse team leaders and nursing assistants excluded from the primary calmness analysis are also central to the emotional reset and leadership modeling components, absorbing additional emotional support and supervisory responsibilities; this study did not measure their workload or burnout, and it remains possible that calmness gains among bedside nurses partly reflect a redistribution of cognitive and emotional load to this excluded group rather than a net systemic improvement. Future evaluations should measure team-leader-level outcomes directly. Twelfth, this study did not measure nurse-to-patient staffing ratio or shift-level workload intensity as covariates; the bundle's effect in ICUs with different staffing models or higher workload burden remains unknown, limiting generalizability beyond units with staffing comparable to GEM's 20-bed ICU.

Thirteenth, the staff questionnaire did not capture administrative and operational factors known to influence ICU work atmosphere, including bed occupancy pressure, admission/discharge turnover frequency, and census variability during the study period, which may have independently influenced perceived calmness and work satisfaction regardless of the CALMER bundle's effect. Fourteenth, the implementation-fidelity audit reported in this manuscript covers only a later eight-week window (May 7 to June 30, 2026) rather than the full six-month study period; pre-implementation and early-phase fidelity data are not available, so compliance trends during the initial adoption phase cannot be characterized.

Fifteenth, the principal investigator provided verbal clarification of item meaning in Tamil when requested, on an unscripted basis; this introduces potential response variability not captured by the standardized English instrument, compounding the administration-related biases described above.

Sustainability and spread

The bundle was designed to support sustainability through the reinforcement component, comprising daily visual cues, peer accountability, and the CALMER champions mechanism. The staff-first (WIIFM) implementation philosophy and safe-to-fail approach were intended to reduce adoption resistance and encourage staff ownership of the cultural shift. However, the study did not directly measure these effects. The intervention's no-capital-expenditure nature is a particular strength in Indian tertiary care ICUs. The CALMER bundle has continued beyond the formal study period and remains operational at GEM Hospital ICU at the time of writing, supporting its operational feasibility; longer-term sustainability of behavioral change requires prospective evaluation. Later-phase fidelity auditing identified emotional reset and mindful workflow and equipment handling as the domains with comparatively lower compliance, indicating these should be prioritized in ongoing team-leader-led reinforcement efforts to sustain the cultural gains achieved during the initial implementation period.

Future directions

This study suggests several directions for future research. First, multicenter validation across diverse Indian ICU settings is needed, with data collection conducted by staff independent of the intervention's design and implementation to reduce the social desirability and administration bias identified in this study's limitations. Second, future studies should incorporate objective acoustic measurement alongside perception-based outcomes. Third, future studies should include patient-specific clinical outcomes, including ICU length of stay, incidence of ICU-acquired delirium, and patient-reported outcome measures, as primary endpoints. Fourth, future studies should use a formally validated ICU calmness perception instrument, subjected to full psychometric evaluation, including confirmatory factor analysis.

## Conclusions

The CALMER bundle, a no-capital-expenditure, six-component, behavior-based quality improvement intervention, was implemented with complete staff orientation in a 20-bed tertiary ICU and was associated with large, statistically significant improvements in perceived ICU calmness across all four assessed domains. Staff perceptions of the work environment improved in parallel, and patient/surrogate perceptions of the ICU environment were strongly positive. The observation that these gains occurred alongside a post-intervention workload rating near the scale midpoint suggests that behavioral reframing of the ICU environment may improve calmness perception through mechanisms not solely dependent on workload reduction; prospective testing with a pre-intervention workload baseline is needed to confirm this. The CALMER framework is feasible, requires no capital expenditure, and is potentially transferable pending multicenter validation, offering a practical model for ICU calmness culture change in Indian tertiary care settings. Multicenter validation with objective noise measurement and patient-specific clinical outcome endpoints represents the logical next step.

## Appendices

Study instruments

This appendix reproduces the full item wording of the four instruments used in this evaluation. Three instruments (Sections A, B, and D) were developed de novo for this study. The fourth (Section C) is a locally adapted version of the NASA Task Load Index (NASA-TLX), rated on a 1-5 scale rather than the original 0-100 format [15]. It is reproduced here as administered and is not presented as an original instrument. Item-to-domain assignments and reverse-scoring flags match those reported in the manuscript's Outcome Measures and Statistical Analysis sections.

Response scale (Sections A, B, and D): 1 = strongly disagree, 2 = disagree, 3 = neutral, 4 = agree, and 5 = strongly agree. Section C (NASA-TLX) uses the same 1-5 numeric scale, anchored with 1 = lowest load/most favorable and 5 = highest load, per item wording.

Staff items in Sections A and B were administered twice per respondent: a retrospective pre-intervention ("before") rating and a post-intervention ("current") rating, as described in Materials and Methods (Data Collection Timeline). Section C was administered post-intervention only. Section D was administered once, cross-sectionally, post-intervention.

Staff questionnaire

Section A - Primary Outcome: Composite ICU Calmness (Q1-Q11)

The instrument comprised 11 items across four domains: environmental calmness, cognitive focus, communication quality, and workflow coordination. The instrument demonstrated good internal consistency, with a Cronbach's alpha of 0.831. Items Q2, Q3, and Q10 were reverse-scored before analysis using the formula score = 6 − raw score on a 1-5 scale. Following reversal, higher scores consistently indicated greater perceived calmness across all 11 items (Table 7).

**Table 7:** Primary ICU calmness questionnaire (11 items) showing full item wording, domain assignment, and scoring ICU: intensive care unit

#	Item as administered	Manuscript ref.	Scoring
1	The ICU environment is calm and well controlled	Environmental calmness (Q1)	As administered
2	Shouting across the ICU is frequent	Environmental calmness (Q2)	Reverse-scored (6 − raw)
3	Noise makes it difficult for me to concentrate on patient care	Cognitive focus (Q3)	Reverse-scored (6 − raw)
4	I am able to work without unnecessary disturbances	Cognitive focus (Q4)	As administered
5	I can focus well on patient management	Cognitive focus (Q5)	As administered
6	Communication among staff is clear and effective	Communication quality (Q6)	As administered
7	Handover occurs without disturbance	Communication quality (Q7)	As administered
8	Equipment handling (trolleys/monitors/ventilators) is done quietly	Environmental calmness (Q8)	As administered
9	Alarm sounds are meaningful and not excessive	Environmental calmness (Q9)	As administered
10	Too many people gather around the bedside during routine care	Workflow coordination (Q10)	Reverse-scored (6 − raw)
11	Overall ICU workflow feels smooth and well coordinated	Workflow coordination (Q11)	As administered

Section B - Secondary Outcome: Staff Work Environment (Q12-Q17)

The work environment assessment comprised six items that evaluated handover quality, handover confidence, freedom from noise-related disruption, work-related stress, workflow disturbance, and overall job satisfaction. Items Q14 and Q15 were reverse-scored before analysis using the formula score = 6 − raw score on a 1-5 scale. Following reversal, higher scores consistently indicated a more favorable work environment across all six items (Table 8).

**Table 8:** Secondary staff work environment questionnaire (6 items) showing full item wording and scoring ICU: intensive care unit

#	Item as administered	Manuscript ref.	Scoring
12	Handover quality in the ICU is good	Handover quality (Q12)	As administered
13	I feel confident after receiving handover	Handover confidence (Q13)	As administered
14	Noise disturbs my work performance	Freedom from noise disruption (Q14)	Reverse-scored (6 − raw)
15	I feel stressed during duty hours	Freedom from work-related stress (Q15)	Reverse-scored (6 − raw)
16	I am satisfied working in this ICU	Job satisfaction (Q16)	As administered
17	I can work without frequent workflow disturbance	Freedom from workflow disturbance (Q17)	As administered

Section C - Secondary Outcome: Workload (Adapted NASA-TLX)

The assessment comprised six domains adapted from the NASA Task Load Index (NASA-TLX) and was not an author-developed instrument. The domains were rated on a locally adapted 1-5 scale rather than the original validated 0-100 format; therefore, the findings are not directly comparable with published NASA-TLX normative data. The assessment was administered post-intervention only to all 40 nursing staff. Table 4 of the manuscript reports the performance domain as a reversed load score, whereas the raw item as administered is presented here. The NASA-TLX is open-source and freely available for use and modification; NASA permission is not required (nasa.gov/human-systems-integration-division/nasa-task-load-index-tlx) (Table 9).

**Table 9:** Adapted NASA-TLX showing full item wording and domains as administered NASA-TLX: NASA Task Load Index

#	Item as administered	Manuscript ref.	Scoring
1	Mental demand is high during duty	Mental demand	As administered
2	Physical demand during duty is high	Physical demand	As administered
3	I feel time pressure during work	Temporal demand	As administered
4	My work requires high effort	Effort	As administered
5	I feel frustrated during duty	Frustration	As administered
6	I am satisfied with my performance	Performance	Reverse-scored for display (6 − raw); raw item shown here

Patient/surrogate questionnaire

Section D - Post-Intervention Environmental Perception (5 Items)

Five items were assessed cross-sectionally post-intervention only among 100 patients or their surrogates. The items evaluated patients’ or surrogates’ perceptions of the ICU environment (Table 10), corresponding to Table 5 of the manuscript.

**Table 10:** Patient/surrogate post-intervention environmental perception questionnaire (5 items) showing full item wording ICU: intensive care unit

#	Item as administered	Manuscript ref.	Scoring
1	The ICU environment felt calm and organized	Calm and organized ICU environment	As administered
2	Staff communication was clear and reassuring	Clear and reassuring communication	As administered
3	I had confidence in the care provided	Confidence in care received	As administered
4	Staff were responsive when needed	Staff responsiveness	As administered
5	Overall ICU experience was satisfactory	Overall ICU experience	As administered
